# Methylation marks breast cancer metastasis: The roles of m^6^A-modified miRNAs and lncRNAs

**DOI:** 10.1016/j.jbc.2026.113127

**Published:** 2026-05-06

**Authors:** Brock A. Humphries, Zhishan Wang, Chengfeng Yang

**Affiliations:** 1 Center for Molecular Imaging, Department of Radiology, University of Michigan, Ann Arbor, Michigan, USA; 2 Stony Brook Cancer Center, Stony Brook University, New York, USA; 3 Department of Pathology, Renaissance School of Medicine, Stony Brook University, Stony Brook, New York, USA

**Keywords:** breast cancer metastasis, epitranscriptomics, long noncoding RNA, microRNA, RNA m^6^A methylation

## Abstract

Breast cancer is the most commonly diagnosed cancer and a leading cause of death in women, with metastasis accounting for most of these fatalities. The mechanism of breast cancer metastasis has not been well understood. Recent research shows that chemical modifications added to RNA molecules after they are made, specifically the addition of a methyl group on the N^6^ position of an adenosine (N^6^-methyladenosine), play a crucial role in how cancer develops, adapts, and resists therapy. This review covers recent advances in understanding how these modifications regulate two major groups of regulatory RNAs, microRNAs, and long noncoding RNAs. Our review highlights that methylation can alter the production, stability, and activity of these RNAs specifically in the context of breast cancer, influencing cell growth, migration, invasion, and resistance to chemotherapy, all of which are key processes in metastasis. We discuss how changes in RNA methylation, regulated by enzymes that add, remove, or recognize these marks, help breast cancer cells adapt to their environment, evade immune detection, and colonize new tissues. The evidence strongly suggests that RNA methylation and its control of regulatory RNAs drive breast cancer progression and survival. Targeting these pathways may allow for more precise diagnostic tests, risk prediction, and development of new treatments. However, further research is required to unravel how these modifications interact with other cellular processes and to translate these findings into effective therapies.

Breast cancer is the most diagnosed cancer (over 30%) and the leading cause of cancer-related death (around 15%) among women in the US (1). Additionally, women have the highest probability of developing an invasive breast cancer (about 1 in 8) in their lifetime compared to other types of cancer (1). One of the major challenges associated with breast cancer mortality is the development of metastatic disease. Despite advances in early detection and treatment, the five-year survival rate plunges from over 95% for localized disease to approximately 30% for metastatic breast cancer (1). Thus, there is an urgent need to elucidate novel regulatory pathways that drive metastasis, particularly those that involve noncoding RNAs as increasing evidence support their important roles in cancer metastasis. This is crucial for the identification of new biomarkers, improving risk stratification, and developing more effective therapeutic strategies. Among these, dynamic post-transcriptional RNA m^6^A modifications have come to the forefront as potential modulators of metastatic progression. While m^6^A modifications occur in all types of RNA transcripts, this review focuses on the impact of m^6^A modifications of noncoding RNAs especially microRNAs (miRNAs) and long noncoding RNAs (lncRNAs) on breast cancer development and progression (Fig. 1), as well as the outlook for their potential to be explored as therapeutic targets and prognosis prediction factors for breast cancer.

**Figure 1:**
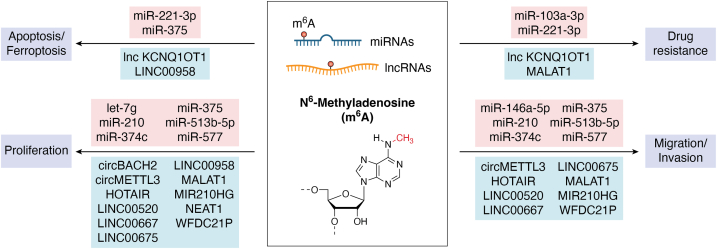
**Regulatory roles of N^6^-methyladenosine (m^6^A) modification on noncoding RNAs and breast cancer phenotypes that underlie metastasis.** This diagram illustrates the diverse functions of m^6^A-marked miRNAs and lncRNAs in the context of breast cancer. Key m^6^A-modified RNAs are organized according to their involvement in four principal metastatic processes: proliferation (*bottom left*), migration/invasion (*bottomright*), drug resistance (*top right*), and apoptosis/ferroptosis (*top left*). The m^6^A modification influences the stability, biogenesis, and regulatory actions of these noncoding RNAs, orchestrating complex gene expression networks that promote breast cancer progression and metastasis. lncRNA, long noncoding RNA; miRNA, microRNA.

## The RNA m^6^A modification machinery

Epitranscriptomics, which is the study of chemical modifications on RNA molecules that regulate their fate and function, has emerged as an essential layer of gene expression regulation, expanding our understanding beyond genomics and epigenetics. More than 170 distinct chemical modifications have been identified on cellular RNAs, affecting their structure, processing, stability, and interactions (2, 3, 4). Among these modifications, N^6^-methyladenosine (m^6^A), the addition of a methyl group on the N^6^ position of an adenosine, is the most prevalent and dynamically regulated internal modification in mammalian coding and noncoding RNAs, including miRNAs and lncRNAs, although other RNA methylations have also been described (5, 6, 7). In mammalian RNA, m^6^A modifications account for approximately 0.1 to 0.4% of nucleotides, with an average of three to five m^6^A sites per transcript (8, 9, 10). These m^6^A modifications can control symmetric, but not asymmetric, commitment in hematopoietic stem cells through m^6^A-tagged transcripts (11), and these marks can persist throughout developmental stages, and in some cases, across generations (12, 13). Functioning as molecular switches, m^6^A and other dynamic modifications fine-tune gene expression in response to environmental stimuli and cellular stress and are increasingly recognized as key regulators of cancer metastasis.

The m^6^A modification is most commonly added in conserved RRACH motifs (where R = A/G, H = A/C/U; or more broadly DRACH, where D = A/G/U), with GGACU being one of the most frequent methylation sites (14). While early studies found m^6^A methylation mainly localized to the 3′ untranslated regions (3′ UTRs) proximal to stop codons (15), subsequent work by the same group and others later identified significant methylation in 5′ UTRs and coding regions (16, 17). Because m^6^A marks are broadly distributed across the transcriptome, they regulate virtually all steps of RNA biogenesis and function, including alternative splicing (18, 19, 20), localization (21, 22), translation efficiency (17, 23), stability (24, 25, 26), and protein synthesis (27, 28). The m^6^A modification is orchestrated by a set of enzymes and RNA-binding proteins collectively known as the m^6^A "writers," "erasers," and "readers." These factors act together to add, remove, and interpret m^6^A marks, thus guiding RNA fate and function through these interconnected processes.

## “Writers”: The m^6^A methyltransferase complex

The m^6^A modification is added to RNAs by a multicomponent methyltransferase complex (“writers”). The catalytic core of this complex is formed by methyltransferase like 3 (METTL3) and 14 (METTL14), which function together as a heterodimer. METTL3 acts as the active methyltransferase, while METTL14 supports substrate recognition and structural stability (29, 30). To enhance specificity of the m^6^A modification, this complex is recruited by factors, such as H3K36me3 (31), or associates with various other secondary components described below.

### Canonical “writers”

In addition to the catalytic core formed by METTL3 and METTL14, the m^6^A writer complex includes several regulatory subunits that lack intrinsic methyltransferase activity but are essential for directing, scaffolding, and localizing the complex to target RNAs.

Wilms tumor 1–associated protein (WTAP) serves as an essential regulatory subunit. WTAP ensures proper localization of the METTL3–METTL14 complex to nuclear speckles where splicing and other RNA processing events occur (32, 33, 34). In breast cancer, WTAP overexpression is generally associated with poorer prognosis (35) and promotes several prometastatic cell behaviors, including cell cycle progression (36, 37), glycolytic metabolism (38), and chemoresistance (39).

Vir-like M6A methyltransferase associated (VIRMA, also known as KIAA1429) serves as a scaffold protein and guides preferential methylation in the 3′ UTR and near stop codons, as well as promoting alternative polyadenylation (34, 40). VIRMA/KIAA1429 is typically overexpressed in breast cancer, where it drives proliferation, invasion, and metastasis through m^6^A-dependent mechanisms (41, 42).

RNA-binding motif protein 15 and 15B recruit the writer complex to specific RNA sites, such as those in XIST, regulating lncRNA-mediated transcriptional silencing (43). Increased expression in breast cancer has been shown to promote proliferation, migration, invasion, and lymphocyte immunity, as well as shift serine and glycine metabolism (44, 45, 46).

Zinc finger CCCH-type containing 13 is considered a tumor suppressor, and its expression is typically lost in breast cancer (47, 48). It has been shown to modulate the nuclear localization by helping WTAP bind the METTL3–METTL14 methyltransferase complex at nuclear speckles and improves the activity of the complex (49, 50), and its loss impairs self-renewal (50).

Beyond their roles in methylation placement, m^6^A writers directly shape ncRNA maturation through structural remodeling and recruitment of processing machinery. METTL3-mediated methylation of primary miRNA (pri-miRNAs) at defined stem–loop regions enhances recognition by DGCR8, promoting Drosha cleavage and accelerating miRNA biogenesis (51). This occurs because m^6^A destabilizes local RNA helices, exposing DGCR8-binding motifs and increasing microprocessor affinity. Similarly, m^6^A deposition on lncRNAs alters their secondary structure to expose or occlude RBP-binding sites, thereby modulating interactions with chromatin modifiers, splicing factors, or decay complexes. VIRMA-directed 3′-UTR methylation further influences lncRNA stability and subcellular localization (41), while RBM15-mediated recruitment of the writer complex to structured lncRNAs such as XIST enables methylation-dependent assembly of silencing complexes (43). Together, these mechanisms illustrate that writers do not simply “mark” RNAs but actively rewire ncRNA processing and function.

### Other “writers”—specialized methyltransferases

Although the primary focus of this review is on m^6^A writers and their roles in regulating miRNA and lncRNA function, we also include a brief overview of additional m^6^A methyltransferases (and related RNA methyltransferases) that act on noncoding RNAs or participate in broader RNA modification networks. Understanding these specialized enzymes, such as methyltransferase like 16 (METTL16), methyltransferase like 5, zinc finger CCHC-type-containing 4, and NOP2/Sun RNA methyltransferase 2 (NSun2) provides a more comprehensive context for how RNA modifications can influence gene expression, splicing, and translational control, processes intrinsically linked to miRNA and lncRNA pathways in cancer biology. Moreover, emerging evidence points to functional interplay and coregulation between these methyltransferases and canonical m^6^A writers, potentially impacting the global noncoding RNA landscape in breast cancer.

In addition, other methyltransferases exist that have distinct RNA methylation substrates. One example is METTL16, a more recently discovered enzyme that acts independently of the METTL3–METTL14 complex (52, 53, 54). METTL16 primarily catalyzes m^6^A modifications at the 3′ UTR of messenger RNA (mRNA) and at adenosine 43 within the conserved “ACAGAGA” motif of U6 small nuclear RNA, which is essential for spliceosome assembly during early U6 small nuclear ribonucleoprotein biogenesis. The methylated adenosine 43 in U6 small nuclear RNA contributes to base pairing at the 5′ splice site of pre-mRNA, suggesting METTL16 has a key regulatory role in splicing and transcript processing (52, 53, 54). Notably, METTL16 is upregulated in breast cancer and has been implicated in the promotion of aggressive behaviors including proliferation, migration/invasion, and ferroptosis (55, 56).

Furthermore, methyltransferase like 5 catalyzes the specific m^6^A methylation of adenosine at position 1832 (A_1832_) located within the 18S ribosomal RNA (57, 58), and zinc finger CCHC-type-containing 4 (ZCCHC4) catalyzes m^6^A methylation on A_4220_ on 28S ribosomal RNA (58, 59). These studies link m^6^A-mediated regulation of ribosome biogenesis to translational control and, most notably, promoting breast cancer cell growth (60).

Finally, NSun2 is a 5-methylcytosine methyltransferase. However, it is important to note that m^6^A methylation by the METTL3–METTL14 complex has been shown to facilitate 5-methylcytosine methylation by NSun2 and *vice versa* (61, 62), suggesting coordination between methyltransferases and/or the methylation marks themselves.

## Erasers: m^6^A demethylases

A hallmark of m^6^A RNA methylation is its dynamic and reversible nature, enabled by a dedicated group of demethylase enzymes known as “erasers.” The discovery that m^6^A marks could be actively removed transformed our understanding of how RNA modifications participate in rapid cellular responses and regulatory circuits. To date, fat mass and obesity-associated protein (FTO) and AlkB homolog 5, RNA demethylase (ALKBH5) are the two principal m^6^A demethylases identified in mammals.

### Fat mass and obesity-associated protein

FTO was the first demethylase shown to act on m^6^A in nuclear RNA. Initially associated with energy homeostasis and obesity, FTO was later found to catalyze oxidative demethylation of m^6^A within mRNA, thus facilitating the removal of methyl groups and affecting RNA metabolism (63). Notably, FTO localizes to both the nucleus and cytoplasm (64), enabling it to regulate m^6^A methylation events at multiple stages of RNA processing and metabolism. FTO’s activity is context-dependent because it is capable of demethylating not only m^6^A but also methylated adenosine adjacent to the 5′ cap structure (m^6^A_m_) (65). Emerging evidence links aberrant FTO expression and function with various aspects of breast cancer biology, including risk, cell proliferation, migration, invasion, and chemoresistance (66, 67, 68, 69, 70, 71, 72, 73), by altering the stability and translation of key oncogenes and tumor suppressors.

### AlkB homolog 5, RNA demethylase

ALKBH5 is the second identified m^6^A demethylase and exhibits specificity for internal m^6^A residues within RNA (74). ALKBH5 localizes predominantly to the nucleus and nuclear speckles (74, 75), and ALKBH5-mediated demethylation influences numerous steps of RNA processing by modulating the fate of both coding and noncoding RNAs. In fact, loss of ALKBH5 can accelerate the export of target mRNAs from the nucleus to the cytoplasm (74), which is reversed by stably re-expressing ALKBH5, suggesting a key role of ALKBH5 in this process. In breast cancer, ALKBH5 supports hypoxia-induced stemness and self-renewal by promoting the demethylation and stabilization of stem cell transcripts (76).

Importantly, the reversibility enabled by erasers allows for rapid and context-specific remodeling of the epitranscriptome. This tunable modification landscape is essential for fine-tuning RNA fate in development and disease, offering promising avenues for therapeutic intervention by targeting the regulatory circuits governed by m^6^A demethylases.

## Readers: Methyl recognition proteins

The functional consequences of m^6^A RNA methylation are ultimately determined by “reader” proteins [methyl recognition proteins (MRPs)] that specifically bind to m^6^A-modified RNAs and translate chemical marks into diverse biological outcomes. These readers orchestrate essential processes in RNA metabolism, including splicing, export, stability, translation, and decay, under physiological and disease conditions.

### YT521-B homology family

The best characterized group of m^6^A readers are the YT521-B homology (YTH) domain-containing proteins, including both cytoplasmic YTH m^6^A RNA-binding protein 1 to 3 (YTHDF1-3) and nuclear YTH domain-containing 11 and 2 (YTHDC1 and YTHDC2) family members. All family members possess a conserved C-terminal YTH domain that bind m^6^A-modified RNA (77), though subtle variations in key tryptophan residues shape their binding specificity and function. For instance, these residues differ among YTHDC1, YTHDF2, and YTHDF1 (78, 79, 80), influencing m^6^A recognition and selectivity. Additionally, their generally unstructured N terminus may support protein–protein interactions or phase separation (81).

Once bound to m^6^A, the cytoplasmic YTHDF proteins (YTHDF1–3 and YTHDC2) play pivotal roles in modulating the fate of m^6^A-modified RNAs. YTHDF1 enhances translation efficiency of methylated transcripts by recruiting ribosome assembly components and initiation factors, supporting rapid protein synthesis in response to cellular stress, growth signals, or oncogenic stimulation (82, 83, 84). YTHDF2, in contrast, is primarily responsible for promoting the degradation of m^6^A-marked mRNAs, thereby determining transcript half-life and enforcing proper gene expression turnover (24, 26, 85). The YTHDF2-mediated decay mechanism generally involves two main, often parallel, pathways (86): (i) directly recruiting the CCR4-NOT deadenylase complex to m^6^A-modified sites for poly(A) shortening and exosome-mediated degradation or (ii) by collaborating with the endoribonuclease HRSP12 to recruit the RNase P/MRP complex for internal endoribonucleolytic cleavage of the transcript. Finally, YTHDF3 serves as a coordinator or handoff factor, fine-tuning the balance between translation and decay, often by interacting with both YTHDF1- and YTHDF2-modified targets (85, 87, 88). YTHDC2 has also been implicated in regulating mRNA stability and translation (89, 90).

Conversely, nuclear YTHDC1 possesses the YTH binding domain but localizes to the nucleus, where it governs RNA splicing, export, and processing. YTHDC1 mediates m^6^A-dependent exon inclusion, alternative splicing patterns, and even the export of methylated RNAs from the nucleus, contributing to finely tuned transcript diversity and protein output (21, 43, 91, 92). YTHDC1 has been linked to regulation of lncRNAs such as XIST, influencing transcriptional silencing and higher-order chromatin organization (43).

Mechanistically, m^6^A readers determine ncRNA fate by recruiting effector complexes that execute decay, stabilization, or translational control. YTHDF2 binding to m^6^A-modified lncRNAs recruits the CCR4–NOT deadenylase complex (26), accelerating transcript decay and reshaping the abundance of metastasis-associated lncRNAs. In contrast, IGF2BP1/2/3 stabilize m^6^A-modified oncogenic lncRNAs by shielding them from deadenylation and recruiting ELAVL1/HuR (93, 94), thereby sustaining prometastatic transcriptional programs. For miRNAs, HNRNPA2B1 recognizes m^6^A-marked pri-miRNAs and enhances DGCR8 recruitment (95), increasing microprocessor activity and boosting mature miRNA output. This reader-mediated control of ncRNA processing and stability provides a direct mechanistic link between m^6^A deposition and the expression of metastasis-promoting miRNAs and lncRNAs.

### Heterogeneous nuclear ribonucleoproteins family

Beyond YTH domain proteins, heterogeneous nuclear ribonucleoproteins (HNRNPs) represent a second important class of m^6^A readers with critical roles in RNA processing and gene regulation. HNRNPs are a large family of RNA-binding proteins that influence various steps of RNA metabolism, including splicing, export, stability, and translation, often by interacting with specific RNA motifs or structures. Key members, heterogeneous nuclear ribonucleoprotein A2/B1 (HNRNPA2B1) and heterogeneous nuclear ribonucleoprotein C (HNRPC), have been shown to directly bind m^6^A-modified RNAs in the nucleus, mediating transcript stability, alternative splicing events, and the nuclear export of transcripts (95, 96, 97, 98, 99, 100). Notably, HNRNPA2B1 and HNRNPC have been shown to facilitate the maturation of ncRNAs by recognizing m^6^A marks, thus promoting efficient processing through the microprocessor complex and modulating overall miRNA abundance (95, 101). However, it should be noted that it has been hypothesized that HNRNPA2B1 mediates effects of m^6^A through an “m^6^A switch” mechanism instead of acting as a direct reader of m^6^A modifications (102). The “m^6^A switch” refers to the phenomenon whereby the addition of m^6^A disrupts local RNA secondary structure, leading to the exposure or masking of specific sequence motifs. This structural remodeling can enhance or inhibit the binding of proteins like HNRNPA2B1 or HNRNPC to their target transcripts, thereby regulating splicing, stability, and processing of both coding and noncoding RNAs in a methylation-dependent manner (101).

### Other “readers”

In addition to the canonical YTH domain proteins and HNRNPs, a growing number of additional m^6^A readers with specialized functions have been identified, which significantly broadens the functional impact of the m^6^A modification in gene regulation and cancer. For example, the insulin-like growth factor 2 mRNA-binding proteins 1 to 3 (IGF2BP1, IGF2BP2, IGF2BP3) have recently been shown to bind methylated RNA through distinct RNA recognition motif and hnRNP-K homology domains (103). Functionally, IGF2BP proteins bind and stabilize their target mRNAs and enhance their translation (93, 104), even under cellular stress or in metastatic contexts. For example, IGF2BP recognition of m^6^A-marked transcripts promotes sustained expression of oncogenes and factors involved in proliferation, stemness, and therapy resistance, linked to breast cancer progression (93, 105). Furthermore, IGF2BP proteins have been proposed as novel stratifying markers and prognostic biomarkers in breast cancer types (106, 107).

The eukaryotic initiation factor 3 (EIF3) complex provides another key link between m^6^A methylation and translational control. EIF3 binds m^6^A-modified 5′ UTRs of specific transcripts and promotes cap-independent translation initiation during cellular stress, viral infection, or in cancer cells with abnormal signaling, which is critical for protein synthesis when canonical cap-dependent translation is inhibited (16, 23). This alternative mode of translation can support the selective expression of stress–response proteins or oncogenes, and aberrant EIF3 activity is increasingly recognized in breast cancer initiation and progression (108, 109, 110, 111).

Interestingly, NF-κB activating protein is a more recently identified m^6^A reader implicated in post-transcriptional gene regulation. NF-κB activating protein recognizes m^6^A-modified RNA to influence mRNA stability and decay and has been linked to regulation of pri-miRNA splicing (112) and ferroptosis (113), both of which are central players in cancer cell biology.

Collectively, many m^6^A readers not only interpret RNA methylation patterns but also act as molecular scaffolds by recruiting additional effector proteins to methylated RNAs. Through their context-dependent specificity and protein interactions, m^6^A readers dictate the cellular fate of thousands of transcripts. Dysregulation or aberrant reader activity can tip the balance between gene activation and repression, driving cancer cell plasticity, therapeutic resistance, and metastasis. Understanding the diversity and mechanisms of m^6^A readers continues to open new windows into RNA-centered regulation and its implications for breast cancer biology. However, more work is needed to elucidate the specific protein complexes and pathways recruited by different m^6^A readers to fully understand how these readers exert their varied cellular functions.

## Molecular features conferring oncogenic transcript specificity

Within a cancer cell, m^6^A deposition is selective not because DRACH (where D = A/G/U, R = A/G, H = A/C/U) motifs are rare, but because writer access and recruitment are rate-limiting. Although DRACH motifs are widespread and m^6^A sites often cluster in coding regions and 3′-UTRs (114), only a subset of motifs is methylated due to transcript-specific differences in (i) cotranscriptional recruitment of the m^6^A writers, (ii) local RNA structure/accessibility, and (iii) downstream reader competition (34, 114). A major source of specificity is locus- and promoter-coupled recruitment, in which transcription factors of chromatin-associated adaptors tether METTL3 to particular genes, thereby biasing methylation toward transcripts produced from these loci. For example, in AML, CEBPZ recruits METTL3 to defined promoters, driving cotranscriptional methylation of targets such as SP1/SP2 while sparing other DRACH-bearing transcripts (115). In parallel, RNA folding and RBP occupancy gate methylation by exposing DRACH motifs in accessible loops or occluding them through protein binding [*i.e.,* structured domains in lncRNAs (*e.g.,* MALAT1 and XIST (28, 43, 116, 117)] exemplify how defined architectures and recruiter RBPs can concentrate the writer complex on particular RNAs. Cancer cells further sharpen selectivity through isoform choice, as alternative polyadenylation and 3′UTR remodeling could create or remove DRACH clusters and nearby AU/CA-rich elements, altering both methylation density and reader engagement on a transcript-by-transcript basis. This bias is further reflected in the intrinsic distribution of m^6^A across the transcriptome, as transcripts encoding housekeeping genes (such as ribosomal proteins) are consistently depleted of m^6^A (15, 34, 118), whereas regulated, context-dependent transcripts, the category to which oncogenes disproportionately belong, are preferentially methylated (34, 114), suggesting that the m^6^A machinery is constitutively oriented away from stable essential transcripts and toward transcripts whose expression is subject to dynamic control. Finally, functional specificity is amplified postmethylation. For example, m^6^A sites positioned near AU-rich or CA-rich elements form composite platforms for stabilizing readers such as insulin-like growth factor-binding proteins (119), and because reader availability and subcellular localization are limiting, only transcripts with the appropriate combination of m^6^A topology and auxiliary motifs exhibit preferential stabilization/translation. Together, these recruitment-, accessibility-, isoform-, and reader-dependent filters explain how the m^6^A machinery preferentially enhances specific protumorigenic RNAs over other transcripts present in the same cell.

Critically, these filters are not passively applied across the transcriptome but are actively co-opted by the oncogenic state itself, biasing m^6^A deposition specifically toward protumorigenic transcripts rather than distributing methylation randomly among accessible DRACH motifs. Glioblastoma stem cells actively redistribute the m^6^A landscape relative to nonneoplastic neural stem cells, with specific m^6^A peaks gained on glioblastoma stem cell transcripts, demonstrating that oncogenic transformation itself reshapes writer recruitment toward protumorigenic RNAs (120). Moreover, compared with thymocytes, T-ALL shows selective m^6^A hypermethylation and HNRNPC binding on transcripts belonging to oncogenic MYC and metabolic networks, with matched reductions in these targets upon METTL3 inhibition, FTO inhibition, or HNRNPC silencing (121), supporting preferential regulation of oncogenic outputs over unrelated transcript classes. This is further exemplified by SRSF7, which is upregulated in cancer and associated with poor prognosis. Mechanistically, SRSF7 physically, and selectively, recruits METTL3/METTL14/WTAP to its binding sites on transcripts involved in cell proliferation, migration, and cancer-related pathways, while SRSF7-binding sites not colocalized with m^6^A peaks are enriched for distinct, nononcogenic transcripts, demonstrating that a cancer-upregulated RNA binding protein can function as a specificity factor that redirects the writer complex toward a defined protumorigenic transcript (122).Tumor-intrinsic stress conditions further enforce this oncogenic bias, as hypoxia-driven reprogramming of the m^6^A epitranscriptome selectively upregulates methylation and expression of a transcript cluster enriched in oncogenic PI3K-Akt, MAPK, and Ras signaling pathways, while other transcript clusters show no such enrichment (123), actively directing m^6^A deposition toward protumorigenic outputs. Notably, this oncogenic bias in m^6^A deposition is established early in the carcinogenic process. Transcriptome-wide profiling of chemical carcinogen-induced cellular transformation across multiple cell types and carcinogens identified dynamic m^6^A changes enriched at cancer-promoting genes, with the oncogene CDCP1 consistently gaining m^6^A modification across all transformation models (124), demonstrating that the m^6^A machinery is redirected toward protumorigenic transcripts even during the initiation of malignant transformation. Together, these findings demonstrate that oncogenic transformation (whether driven by cell-intrinsic reprogramming, cancer-upregulated RNA binding proteins, microenvironmental stress, or carcinogen exposure) converges on the mechanism of selective redirection of the m^6^A machinery toward protumorigenic transcripts over the other RNAs present in the same cell.

## Stage-, subtype-, and microenvironment-dependent roles of m^6^A in breast cancer

Recent research demonstrates that patterns of m^6^A modifications differ substantially between breast cancer subtypes. For example, Yang *et al.* found that m^6^A methylation patterns not only distinguish breast cancer subtypes, particularly basal-like from luminal, but also identify biologically and clinically distinct subgroups within the luminal subtype. Notably, luminal breast cancer samples with m^6^A methylation profiles resembling those of basal-like tumors were associated with poorer overall outcomes (106). These methylation-driven differences impact immune activity and tumor progression, providing a refined method for prognosis and highlighting new stratification strategies for personalized therapy. Recent epitranscriptomic profiling has revealed that m^6^A methylation patterns vary markedly between breast cancer subtypes, with triple-negative breast cancer (TNBC) exhibiting widespread m^6^A methylation of genes involved in oncogenic signaling and invasion (125). In contrast, luminal subtypes display distinct m^6^A modification signatures from TNBC, highlighting the potential of m^6^A methylation levels as novel biomarkers for subtype classification and patient prognosis (125). In addition, Ouyang *et al.* also found that m^6^A levels are significantly downregulated after chemotherapy in hormone receptor-positive breast cancers, but they remain stable in TNBC, suggesting fundamental differences in epitranscriptomic regulation and treatment response between subtypes (126). Complementary work by Dorgham *et al*. demonstrated that the effects of m^6^A modification on cell proliferation and migration are stage specific (127). Specifically, in early-stage, nontumorigenic MCF10A cells, reducing m^6^A levels increase proliferation but decreases migration. In contrast, in more advanced, malignant MCF10AT1 and MCF10CA1h cells, lowering m^6^A levels promotes migration and decreases proliferation. Notably, these late-stage cells exhibit an inherent resistance to changes in m^6^A levels (127), suggesting that adaptation or alternative regulatory mechanisms may be at play.

In addition, m^6^A-related gene signatures have been shown to stratify breast cancer patients by immune infiltration status and prognosis, providing a framework for predicting immunotherapy response and correlating with tumor microenvironment (TME) characteristics (128). Furthermore, m^6^A methylation patterns, through an “m^6^A score”, reveal breast cancer subtypes with profoundly different immune microenvironments and prognoses. Low m^6^A score (immune-inflamed/basal-like) is associated with active immune infiltration and better immunotherapy response, while high m^6^A score (immune-excluded/luminal A) corresponds to stromal activation and better overall survival (129). Collectively, these findings highlight the dynamic nature of m^6^A modifications across breast cancer subtypes and stages, underscoring its potential as both a biomarker and therapeutic target.

In addition to its subtype and stage-dependent functions, m^6^A has emerged as a pivotal regulator of gene expression programs that drive breast cancer progression, notably through its impact on epithelial-to-mesenchymal transition (EMT). EMT is a biological process in which epithelial cells acquire mesenchymal traits, enhancing their motility, invasiveness, and survival, all of which are key features underlying tumor metastasis (130, 131). Mechanistically, m^6^A influences EMT by modulating stability, splicing, and translation of specific mRNAs and ncRNAs that encode EMT markers and regulators. For example, recent transcriptomic and functional studies have demonstrated that manipulating m^6^A levels leads to changes in breast cancer migration and EMT marker expression such as E-cadherin, N-cadherin, and vimentin, where lower m^6^A levels is often associated with increased migration and enhanced EMT (127, 128). These findings reflect a dynamic regulation of EMT by m^6^A and are further corroborated by gene expression analyses linking m^6^A-dependent control of EMT *via* cell cycle regulators (*i.e.,* CDKN1A (126)), apoptosis (*i.e.,* BAX (126)), and key EMT transcription factors (*i.e.,* ZEB1 (132)), ultimately influencing metastatic potential. Moreover, Zhang *et al.* showed that stratification of breast cancer patients based on m^6^A-related immune signatures correlates with EMT, immune infiltration, and prognosis (128), underscoring m^6^A modification’s role in integrating molecular cues that drive both tumor progression and immune evasion. Together, these studies illustrate that m^6^A is not simply a marker but a dynamic driver of EMT and breast cancer progression, with nuanced effects depending on cell subtype and disease stage.

Recent work highlights the critical role of FTO in this process. Jeschke *et al.* demonstrated that FTO downregulation leads to increased m^6^A methylation on RNA transcripts involved in Wnt/β-catenin signaling pathway (133), an established driver of EMT (134). Mechanistically, loss of FTO promotes EMT by altering the stability of key mRNAs, resulting in enhanced expression of mesenchymal markers, elevated migration, and invasion. Notably, FTO-deficient tumors exhibit increased sensitivity to Wnt pathway inhibitors (133), suggesting m^6^A-mediated regulation not only facilitates EMT but also uncovered therapeutic vulnerabilities.

However, while these studies underscore the importance of m^6^A regulators in EMT and cancer progression, it is critical to recognize that m^6^A acts on a broad array of transcripts, in both malignant and nonmalignant settings. Global targeting of the m^6^A modification machinery (for example, with METTL3 inhibitors) could thus have widespread effects, potentially leading to significant off-target consequences or unanticipated resistance, cancer progression, with nuanced effects depending on cell subtype and disease stage.

In addition to effects on EMT, the changes in the TME have also been shown to influence m^6^A levels in breast cancer, adding an additional layer of complexity to the regulation of gene expression and tumor behavior. For example, hypoxia, a hallmark of cancer progression resulting from rapid tumor growth and poor vascularization, can induce substantial changes in m^6^A modification patterns. Mechanistically, hypoxic stress has been shown to regulate the expression and activity of m^6^A writers, erasers, and readers, thereby modulating overall m^6^A deposition and downstream RNA fate (135). For instance, hypoxia can upregulate ALKBH5, leading to site-specific m^6^A demethylation and stabilization of stemness-related transcripts (76) or enhance METTL3-mediated methylation of oncogenic targets (123) and alter subcellular localization or protein–protein interactions of these enzymes. Importantly, in some contexts, hypoxia-induced shifts in m^6^A methylation may occur independently of changes in METTL3/14 and ALKBH5 expression levels (136), suggesting alternative methyltransferases or regulatory mechanisms may also be involved. Xiong *et al.* found that m^6^A methylation stabilizes RIPOR2, increasing its protein expression and driving tumor growth and metastasis in response to hypoxia (123). Furthermore, Zhang *et al*. found that hypoxia-induced loss of m^6^A stabilizes NANOG, enhancing its expression and the maintenance of cancer stem cells, thereby facilitating breast cancer initiation, metastasis, and therapeutic resistance (76). In terms of stage-specific changes, Fry *et al.* found that stress from hypoxia stimulated an increase in m^6^A levels in both immortalized and transformed cells (136). However, this increase in m^6^A levels led to a more malignant phenotype in transformed cells, but not their immortalized precursors. Finally, Shen *et al.* demonstrated that patterns of m^6^A methylation are closely linked to hypoxia status and immune microenvironment in TNBC, with distinct m^6^A-hypoxia gene signatures defining tumor subgroups that differ markedly in prognosis, immune cell infiltration, and response to immunotherapy (137). By integrating m^6^A modification and hypoxia-related genes, the authors developed a risk score that stratifies TNBC patients and more accurately predicts clinical outcomes and immunotherapy responsiveness, highlighting the TME-epitranscriptomic axis as a key determinant of breast metastatic potential. Taken together, these studies underscore that hypoxia-driven changes in m^6^A methylation are central to shaping the molecular heterogeneity, stemness, immune landscape, and therapeutic susceptibility of breast cancer.

## miRNAs and lncRNAs: Key regulators of gene expression and critical players in metastasis

### miRNAs

miRNAs are a large family of small (20–22 nucleotides) noncoding RNAs that act as post-transcriptional regulators by binding mostly to the 3′UTRs of target mRNAs, inducing degradation or repressing translation. The biogenesis of miRNAs involves a tightly regulated, multistep process. Initially, miRNAs are transcribed in the nucleus by RNA polymerase II as primary transcripts (pri-miRNAs), which are often hundreds to thousands of nucleotides in length and are both polyadenylated and capped (138, 139). These pri-miRNA transcripts are processed by the microprocessor complex, consisting of the type III RNase Drosha and its partner DGCR8, trimming them into approximately 70-nucleotide precursor miRNAs (pre-miRNAs) (140, 141). Pre-miRNAs are then exported to the cytoplasm *via* exportin-5, where another type III RNase, Dicer, further cleaves them to generate a ∼21 to 22 nucleotide miRNA duplex (142, 143). After strand separation, the mature miRNA associates with Argonaute proteins to assemble into the RNA-induced silencing complex, which guides the miRNA to recognize complementary sequences in target mRNAs.

Through this mechanism, miRNAs modulate crucial cellular pathways, including proliferation, apoptosis, migration, and differentiation, which are all processes integral to cancer development and progression. In breast cancer and other malignancies, a disrupted balance between oncogenic miRNAs (oncomiRs) and tumor suppressor miRNAs drives aberrant gene regulation, promoting uncontrolled cell growth, evasion of apoptosis, and acquisition of metastatic traits. For instance, overexpression of oncomiRs, such as miR-21 or the miR-17 to 92a cluster, enhance proliferation and invasion (144, 145, 146), while we and others have shown that loss of tumor suppressor miRNAs, such as let-7, miR-205, or the miR-200 family, fosters EMT and metastatic potential (147, 148, 149, 150, 151). Thus, the biogenesis and function of miRNAs are central to both normal cellular homeostasis and the pathways that underlie tumorigenesis and metastasis.

### LncRNAs

LncRNAs are a diverse class of transcripts (>200 nucleotides in length) that, by definition, lack protein-coding potential and have emerged as central regulators of gene expression in cancer. It is worth noting, however, that some transcripts previously annotated as lncRNAs have been found to encode functional peptides, and in these cases, reclassification is warranted, indicating that they were initially misidentified as lncRNAs (152, 153). However, unlike small noncoding RNAs, lncRNAs exert their functions through a wide range of mechanisms, including (i) direct binding to chromatin to influence accessibility, (ii) acting as scaffolds for protein complexes, (iii) serving as decoys for RNA-binding proteins or miRNAs, and (iv) modulating nuclear architecture or mRNA splicing (154, 155). This versatility enables lncRNAs to integrate multiple signaling pathways and fine-tune gene expression at transcriptional, post-transcriptional, and even post-translational levels.

Unlike miRNAs, lncRNAs are generated through various pathways, greatly impacting processing, shape, localization, stability, and function (156). The major types of lncRNAs include (i) the classical intergenic region-transcribed lncRNAs, which share the same RNA polymerase II transcription and RNA processing as mRNAs, featuring a 5′ m^7^G cap and polyadenylated 3′ tail, splicing, and are either retained or exported from the nucleus through nuclear pore complexes (156), (ii) antisense transcripts of protein-coding genes, and (iii) RNA polymerase II transcript-derived unconventional lncRNAs, where stabilization occurs by RNase P cleavage (157, 158), capped by snoRNA–protein complexes (159, 160), or through covalently closed circular structures [circular RNAs (circRNAs)] (161, 162).

In cancer biology, aberrant expression and activity of lncRNAs are increasingly implicated in tumor initiation, progression, and metastasis. Numerous lncRNAs function as oncogenes or tumor suppressors, and their functional role is determined by specific aspects of their cellular context, including cell type, tissue origin, signaling pathways active in the TME, stage of disease, and/or their interacting partners present within the cell. LncRNAs can regulate EMT, influence stemness and resistance to therapy, or modulate the immune microenvironment (163, 164, 165). In breast cancer, prometastatic lncRNAs such as HOTAIR, HUMT, and TROJAN or the antimetastatic ANCR have been shown to promote or inhibit growth, invasion, migration, and metastatic colonization by reprogramming gene expression networks and interacting with chromatin-remodeling complexes, respectively (166, 167, 168, 169). Specifically, TROJAN contributes to breast cancer cell growth by binding the NF-κB repressor NKRF, preventing repression of the NF-κB transcriptional activator RELA, which leads to the upregulation of CDK2 transcription and promoting cell cycle progression (168). Therefore, understanding how lncRNA expression and epitranscriptomic modulation cooperate to drive breast cancer progression may open new avenues for biomarker discovery and targeted therapeutic intervention.

## Impacts of the m^6^A modification on miRNA biology and functions

### Impact of the m^6^A modification on miRNA biogenesis

**Figure 2:**
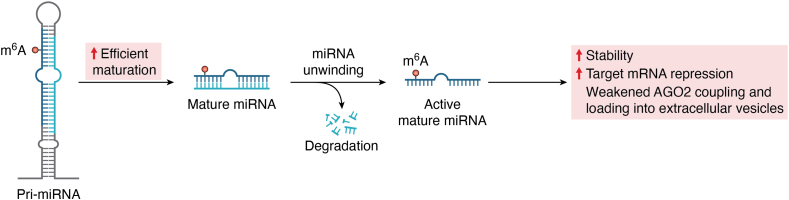
**Schematic overview of m^6^A modification regulating miRNA maturation, stability, and function.** This figure illustrates the key roles of N^6^-methyladenosine (m^6^A) modification (*red circle*) in miRNA processing pathways. m^6^A marking of pri-miRNAs promotes more efficient maturation into active mature miRNAs. In mature miRNAs, m^6^A imparts multiple regulatory effects, including increased stability, enhanced target mRNA repression, and facilitation of export out of the cell *via* extracellular vesicles. Collectively, these processes tightly control miRNA fate and gene regulatory activity in cells. miRNA, microRNA; mRNA, messenger RNA; pri-miRNAs, primary miRNA.

The m^6^A modification functionally reshapes miRNA biogenesis, and this regulatory axis is especially important in breast cancer where metastatic progression depends on tight gene expression control (Fig. 2). Early evidence demonstrated that m^6^A marks deposited on pri-miRNA transcripts facilitate their recognition and processing by the microprocessor complex, specifically promoting the recruitment of DGCR8 (51, 95), a key protein in pri-miRNA cleavage. This modification aids the conversion of pri-miRNAs to pre-miRNAs, accelerating subsequent maturation steps and ensuring efficient miRNA production. While this study shows a positive role for m^6^A in enhancing processing, others report that knocking down “erasers”, such as FTO, can reduce the baseline levels of certain miRNAs without altering overall pri-miRNA abundance (170), suggesting that the m^6^A modification might influence miRNA stability or further processing events.

In breast cancer, the m^6^A modification has been shown to globally reshape the miRNA landscape. For example, multiple studies have shown that upregulation of METTL14 modulates both total m^6^A levels and miR-146a-5p (171) or miR-375 expression (172) in breast cancer cells. Separately, Pan *et al*. showed that METTL3 accelerates maturation of pri-miR-221-3p in MCF-7 breast cancer cells (173). In addition, HNRNPA2B1 influences miRNA maturation by facilitating microprocessor access to m^6^A-marked pri-miRNAs (95) and is also upregulated in endocrine-resistant breast cancer (LCC9) and globally alters the miRNA transcriptome when overexpressed in MCF-7 cells (174). Interestingly, the functional link between m^6^A and miRNA biogenesis is also conserved in plants, as evidenced by studies in *Arabidopsis thaliana*, where mRNA adenosine methylase deposits m^6^A on pri-miRNAs to modulate miRNA processing, highlighting a possibly evolutionary preserved mechanism (175). Together, these findings underscore a central role for m^6^A in modulating efficiency, specificity, and diversity of miRNA production, which is a regulatory axis with important implications for breast cancer progression, drug resistance, and potentially for biomarker development in oncology.

The m^6^A modifications of mature miRNAs have recently been shown to influence their fate in cellular export and intercellular communication. Notably, m^6^A tagging of mature miRNAs can reduce their intracellular gene regulatory activity, instead promoting their sorting into extracellular vesicles and subsequent export from the cell (176). This process allows modified miRNAs to participate in intercellular signaling, acting as messengers within the TME or physiological tissue niches. The m^6^A-driven export mechanism thus not only fine-tunes the intracellular abundance and action of miRNAs but also enables dynamic exchange of regulatory information between neighboring or distant cells. More research is needed to better elucidate epitranscriptomic control over miRNA export and extracellular function. However, these findings highlight an additional layer by which m^6^A shapes gene regulatory networks through RNA-based communication beyond the boundaries of the cell.

### Impact of the m^6^A modification on miRNA stability

Beyond regulating miRNA biogenesis, the m^6^A modification also has direct and complex effects on the stability of mature miRNAs and related noncoding RNAs, contributing to the fine-tuning of post-transcriptional gene regulation in health and disease. Recent evidence demonstrates that m^6^A-marked miRNAs and m^6^A-modified noncoding RNAs can be subjected to endoribonucleolytic cleavage by specific ribonuclease complexes, such as RNase P/MRP, highlighting a novel mechanism of selective miRNA turnover (177). Moreover, in breast cancer cells, m^6^A-mediation is intricately linked to disease progression. In this respect, cadmium-induced increased m^6^A modification of pre-miR-374c-5p interferes with its maturation into the functional miR-374c-5p, ultimately leading to metastasis by derepressing GRM3, a key gene involved in invasive behavior (178). This illustrates a crucial role for m^6^A in controlling miRNA stability as well as cancer cell plasticity in response to environmental stressors.

Additionally, genome-wide mapping of m^6^A methylation on circRNAs reveals that these modifications are widespread and exhibit cell type–specific methylation patterns distinct from those observed on linear mRNAs (179). This m^6^A methylation does not appear to promote degradation of circRNAs themselves, but because circRNAs often act as miRNA sponges or modulators of miRNA activity (180), it is speculated that m^6^A-dependent control of circRNA stability and turnover constitutes another layer of complexity in regulating miRNA stability and cellular response to malignancy. Collectively, these findings underscore the multifaceted ways in which m^6^A methylation can direct miRNA abundance and function, which can affect RNA fate, stress response, and cancer progression through both canonical and noncanonical regulatory routes.

### Impact of the m^6^A modification on miRNA action

Accumulating evidence demonstrates that m^6^A modification of target mRNAs profoundly influences miRNA-mediated gene regulation, adding an additional layer of epitranscriptomic control to post-transcriptional silencing. Transcriptome-wide analyses reveal that m^6^A peaks are frequently colocalized with miRNA binding sites (181), with up to 80% of m^6^A-modified transcripts containing at least one miRNA target region (182). The m^6^A modification can enhance or inhibit miRNA targeting and function, depending on the transcript and cellular context. For example, m^6^A methylation has been shown to promote miRNA-mediated repression of mRNAs by increasing accessibility of binding sites and facilitating recruitment of the RNA-induced silencing complex (181, 183). Spatial correlation between m^6^A and miRNA binding sites is further supported by global mapping studies, which illustrate that m^6^A modification can increase the density and efficacy of miRNA–mRNA interactions (183). Conversely, m^6^A may also impede miRNA action in certain contexts or on specific transcripts, suggesting a dynamic regulatory interplay.

In breast cancer, dysregulated m^6^A deposition has a direct impact on miRNA-guided mRNA repression. For example, high levels of the oncoprotein HBXIP suppress the tumor-suppressive miRNA let-7g, which normally binds the 3′ UTR of METTL3 mRNA to inhibit its translation. This relief of suppression leads to increased METTL3 expression, forming a positive feedback loop in which METTL3 further promotes HBXIP expression *via* m^6^A modification, thereby enhancing cancer progression (184). Similarly, overexpression of METTL14, the other subunit of the methyltransferase catalytic core complex, has also been shown to cause global changes in miRNA expression. Notably, METTL14 specifically enhances the maturation and stability of miR-146a-5p, which in turn contributes to a more aggressive and metastatic breast cancer phenotype (171).

There is also mounting evidence for reciprocal regulation, where miRNAs themselves can modulate the levels of m^6^A methylation on their targets or even influence methyltransferase expression (185). Altogether, these findings underscore the bidirectional and multifaceted relationship between m^6^A marks and miRNA function in gene regulatory networks, with far-reaching implications for cancer biology, cell fate transitions, and therapeutic response.

### Impact of the m^6^A modification on miRNA function in metastasis

**Figure 3:**
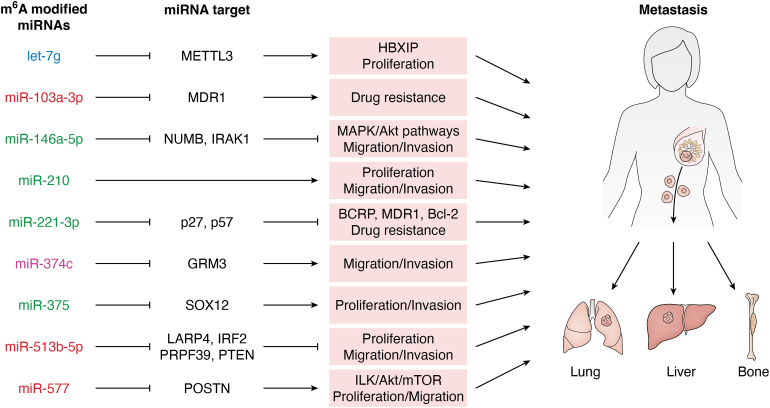
**Schematic representation of m^6^A-modified miRNAs and target genes driving breast cancer metastasis.** The diagram illustrates how specific m^6^A-modified microRNAs (miRNAs), such as let-7g, miR-103a-3p, miR-146a-5p, miR-210, miR-221-3p, miR-374c, miR-375, miR-513b-5p, and miR-577, regulate key genes involved in metastatic progression. These miRNAs target and suppress metastasis-associated genes (METTL3, MDR1, NUMB, IRAK1, CDKN1B, p57, GRM3, SOX12, and POSTN), thereby influencing cellular behaviors. Text over the second arrow within each pathway indicates the process by which a given miRNA affects metastasis, and listed gene is downstream of the miRNA target and impacts the metastatic process. If no gene is shown, it was not defined in the manuscript. In the diagram, color-coding indicates impact of m^6^A modification on miRNA expression or function in cancer cells; where *green* denotes miRNAs stabilized and with accelerated maturation, *red* denotes miRNAs sponged by a competing endogenous RNA (ceRNA), *blue* denotes miRNAs either stabilized by m^6^A or suppressed by an oncoprotein, and *pink* denotes miRNAs whose maturation is interfered with by m^6^A. The exact mechanisms for each miRNA are described in the text. Collectively, the m^6^A-dependent regulation of these gene networks contributes to breast cancer metastasis. m^6^A, N6-methyladenosine; METTL3, methyltransferase like 3.

The m^6^A modification of miRNAs shapes metastatic progression in breast cancer by dynamically regulating the maturation and silencing of key gene targets. A diverse set of m^6^A-modified miRNAs converge on critical pathways controlling migration/invasion, proliferation, and drug resistance, collectively driving metastatic potential (Fig. 3).

### Migration and invasion

Metastatic dissemination is the primary cause of breast cancer mortality, and cellular migration and invasion are foundational to this process. Emerging studies reveal that the m^6^A modification of miRNAs is a central epitranscriptomic mechanism that fine-tunes the migratory phenotype through tightly regulated gene expression networks.

For example, METTL14-mediated m^6^A methylation specifically enhances the maturation and expression of miR-146a-5p, a key pro-migratory miRNA that targets key migratory suppressors NUMB and IRAK1 (186, 187), likely through increased recognition and processing by DGCR8 (51, 95), thereby activating extracellular matrix remodeling and cellular motility in breast cancer cells (171). Likewise, elevated METTL3 activity increases m^6^A deposition on pri-miR-221-3p, resulting in abundant mature miR-221-3p, which inhibits cell adhesion molecules and epithelial markers, promoting cytoskeletal rearrangement, detachment, and tissue barrier penetration, all critical steps in invasive progression (173). Other m^6^A-regulated miRNAs, including as miR-374c, miR-210, and let-7g, have also been shown to contribute to increased cell motility and metastatic colonization in breast cancer models (178, 184, 188), further expanding the list of epitranscriptomic targets involved in breast cancer migration and invasion. However, Zhao *et al.* found that METTL14 can instead inhibit breast cancer cell invasion through the miR-375-*SOX12* axis (172), suggesting a target- and context-dependent role for METTL14 in breast cancer.

The m^6^A modification deposited onto miRNAs can also exert their functions through “reader” proteins such as HNRNPA2B1. This nuclear reader binds m^6^A-marked pri-miRNAs and delivers them to Drosha/DGCR8 for efficient cleavage, maintaining a robust pool of pro-migratory and pro-invasive miRNAs (51). Other m^6^A readers, including YTHDF1 and YTHDF3, have been implicated in regulating miRNA turnover, translation, and even cellular localization, suggesting a more complex network of m^6^A-dependent RNA–protein interactions that shape migratory behavior (79, 87, 176). Furthermore, overexpression of HNRNPA2B1 in breast cancer cells, especially in endocrine-resistant and highly invasive subtypes, further skews the miRNA landscape toward a phenotype that results in enhanced invasion and metastasis (174). This specificity could be achieved because HNRNPA2B1 recognizes and binds m^6^A marks within sequence motifs (such as the DRACH consensus) that are frequently present in the pri-miRNAs of migratory and invasive genes. Moreover, aggressive breast cancer subtypes could produce higher levels of these m^6^A-modified, pro-invasive ncRNAs, making them preferred substrates for HNRNPA2B1-mediated processing and maturation.

It is increasingly apparent that alterations in m^6^A signaling can be influenced by the TME. Signals such as hypoxia, inflammatory cytokines, and extracellular matrix stiffness modulate the expression and activity of m^6^A machinery, leading to dynamic shifts in miRNA-mediated invasion and adaptive responses to stromal and immune cues (137). Under hypoxic conditions, specificity for pro-invasive transcripts and miRNAs is achieved through several mechanisms, such as hypoxia-responsive transcription factors (such as HIF1α) upregulating m^6^A “erasers” like ALKBH5, or RNA secondary structure, local sequence motifs (*e.g.,* DRACH consensus), and the presence of other RNA-binding proteins can guide “writers” to deposit m6A marks on select transcripts (76, 189), favoring those involved in invasion, migration, and metastasis. As a result, hypoxia-induced m^6^A modification stabilizes pro-invasive mRNAs and miRNAs, such as those regulating EMT or matrix remodeling, thereby enhancing the metastatic potential of breast cancer cells (36, 123).

Fundamentally, these alterations can converge on EMT, enhancing their motility, invasiveness, and survival, all of which are key features underlying tumor metastasis (127, 130, 131). In this regard, the m^6^A reader YTHDF3 binds m^6^A-modified ZEB1 mRNA and increases its stability, which is accompanied by decreased E-cadherin and increased N-cadherin/vimentin in TNBC cells (127). Consistent with a broader role for METTL3-dependent m^6^A in EMT programs, METTL3 knockdown is associated with stage-dependent changes in EMT marker expression in a breast cancer progression model (132). These changes support detachment from the primary tumor, invasion into the surrounding stroma, and entry into the circulation. Mechanistically, this may occur through m^6^A-dependent promotion of oncomiR maturation that suppresses epithelial characteristics or by facilitating the degradation of tumor-suppressive miRNAs that maintain the epithelial phenotype (95).

It is important to note that these epitranscriptomic effects can be context-specific, intensifying in advanced-stage and aggressive breast cancer subtypes, and integrating with other networks that sustain migration and invasion. Cutting-edge technologies, such as single-cell and spatial epitranscriptomic profiling, live-cell imaging, and precision RNA modification editing, are expected to further clarify the temporal and spatial mechanisms by which m^6^A-miRNA regulation drives metastatic behavior. The dynamic control exerted by m^6^A modification over miRNA biogenesis, stability, and function highlights the m^6^A-miRNA axis as a master regulator of metastasis.

### Growth and proliferation

Sustained, uncontrolled cell growth is a defining feature of metastatic breast cancer, enabling tumor cells to seed and colonize distant sites. Recent epitranscriptomic studies have revealed that m^6^A methylation plays a central role in promoting this oncogenic proliferation through its dynamic control of miRNA function.

At the core of this regulation are the “writer” enzymes METTL3 and METTL14, which deposit m^6^A marks onto pri-miRNA transcripts, such as pri-miR-375. This modification increases the efficiency of recruitment to the microprocessor complex (Drosha/DGCR8), likely through HNRNPA2B1 binding (51, 95), expediting the biogenesis and maturation of growth-promoting miRNAs (172). Mature miR-375 then suppresses cell cycle inhibitors and apoptosis-related genes, driving enhanced cell division and survival, which are essential for metastatic colonization. In addition to miR-375, other miRNAs such as miR-210, miR-221-3p, and let-7*g* have also been implicated in m^6^A-dependent regulation of breast cancer proliferation. m^6^A-mediated maturation and stabilization of these miRNAs promotes cell cycle progression, metabolic adaptation, and resistance to apoptosis, broadening the spectrum of noncoding RNAs that fuel tumor outgrowth (173, 178, 184).

In addition to miRNA regulation, m^6^A also modifies circRNAs and lncRNAs, allowing them to act as competitive endogenous RNAs (ceRNAs) for miRNAs. These m^6^A-marked circRNAs and lncRNAs can bind and sequester tumor-suppressive miRNAs (such as let-7 and miR-205), which normally inhibit genes responsible for cell cycle progression, metabolic adaptation, and evasion of apoptosis (179, 181, 182, 190, 191). The outcome is release of oncogenic and anti-apoptotic pathways, supporting aggressive and persistent growth even under stressful microenvironmental conditions.

Changes in the activity or expression of m^6^A “writers,” “readers” (such as YTHDF proteins and HNRNPA2B1), or “erasers” (FTO, ALKBH5) disrupt the normal checks on cell proliferation. Overproduction or stabilization of progrowth miRNAs and ceRNAs or accelerated degradation of growth-inhibitory RNAs upsets cell cycle control and fuels growth. Among m^6^A readers, YTHDF1 enhances translation of growth-related transcripts through recruitment of translation initiation factors (23, 192), YTHDF3 coordinates RNA decay through the recruitment of the CCR4-NOT deadenylase complex (26), and IGF2BP proteins protect oncogenic RNAs from degradation through various pathways discussed in more detail later in this review, collectively driving aggressive tumor outgrowth (79, 87, 93). Importantly, IGF2BPs do not exhibit intrinsic selectivity for oncogenic RNAs, rather they bind and stabilize a broad spectrum of m^6^A-modified transcripts, including both oncogenic and tumor suppressive RNAs. Their apparent protumorigenic activity often reflects upregulation of oncogenic targets or context-dependent transcript availability in cancer cells. Functional studies using genetic knockdown or pharmacologic inhibition of METTL3 and METTL14 have demonstrated reduced proliferation *in vitro* and suppression of tumor growth in murine xenograft models, highlighting the direct causal role of m^6^A in breast cancer progression (29, 36). Notably, these m^6^A-driven pathways integrate signals from the TME; *e.g.,* hypoxia-induced changes in m^6^A stabilize transcripts that promote stemness and proliferation (29, 36, 38, 123), further driving tumor aggressiveness and therapy resistance.

Collectively, m^6^A methylation orchestrates a multilayered network of noncoding RNA maturation, competition, and turnover that promotes breast cancer cell proliferation, supporting metastatic growth and clinical relapse. These mechanistic insights underscore m^6^A and its regulated RNA substrates as promising targets for interventions aimed at limiting tumor expansion and improving outcomes in metastatic breast cancer.

### Drug resistance

Drug resistance remains one of the greatest challenges in the treatment of metastatic breast cancer, frequently leading to disease relapse and limiting chemotherapeutic success. Recent discoveries highlight the pivotal role of m^6^A methylation in the regulation of miRNAs that drive the emergence of drug-resistant tumor phenotypes.

At the core of this epitranscriptomic regulation is the m^6^A "writer" METTL3, which intensifies methylation on pri-miR-221-3p and accelerates its processing into mature miR-221-3p (173). Elevated miR-221-3p levels hinder chemotherapy-induced apoptosis by suppressing key cell cycle inhibitors (such as CDKN1B/p27 (193) and p57 (194)) and activating prosurvival gene networks. This adaptation allows cancer cells to persist and proliferate in the presence of cytotoxic drugs like adriamycin.

Studies have shown that m^6^A methylation plays a key role in drug transporter regulation by modifying both miR-103a-3p and the lncRNA KCNQ1OT1 (195). Zhou *et al.* demonstrated that in doxorubicin-resistant cells, METTL3 mediates m^6^A modification of lnc KCNQ1OT1. Through combining RNAi and actinomycin D treatment, they found that METTL3 expression is positively correlated with lnc KCNQ1OT1 stability, where higher METTL3 levels reduce its degradation. As a result, METTL3-mediated m^6^A methylation stabilizes lnc KCNQ1OT1, bolstering its function as a molecular sponge for miR-103a-3p. Supporting this, knockdown of lnc KCNQ1OT1 increased the expression of miR-103a-3p (195). Reduced activity of miR-103a-3p subsequently leads to increased MDR1 (ABCB1) expression, a crucial drug efflux pump that removes chemotherapeutic agents from cancer cells, thereby decreasing intracellular drug accumulation and contributing to multidrug resistance (195).

In addition, the m^6^A mark plays a direct role in a feedback loop involving the lncRNA DLGAP1-AS1, miR-299-3p, and the m^6^A writer WTAP (39). Mechanistically, WTAP-mediated m^6^A methylation of DLGAP-AS1, potentially through the recruitment of specific m^6^A reader proteins such as IGF2BP1/2 (196), leads to stabilization of the lncRNA by protecting it from RNase-mediated degradation. This increased stability enables DLGAP1-AS1 to act as a sponge for miR-299-3p, thereby preventing miR-299-3p-mediated suppression of WTAP. When m^6^A-mediated stabilization is lost, DLGAP1-AS1 is degraded, allowing miR-299-3p to target WTAP mRNA, which reduces WTAP activity and adds an additional layer of complexity to the regulatory circuit. Importantly, this feedback circuit enhances global m^6^A methylation (39), further amplifying proresistance gene networks through coordinated regulation of both coding and noncoding RNAs.

Collectively, these mechanisms underscore how m^6^A modification and its regulated noncoding RNAs generate and reinforce complex networks that support drug resistance by modulating cell death, drug efflux, and survival pathways. The intricate crosstalk between m^6^A “writers,” “readers,” and “erasers” can rapidly remodel gene expression in response to chemotherapy, fostering adaptive phenotypes that evade cytotoxic stress. However, it is important to note that the effects of m^6^A modification on drug resistance are highly context-dependent, with cell heterogeneity, tumor subtype, and compensatory epitranscriptomic pathways possibly influencing outcomes. Further studies are needed to untangle the redundancy and interplay among m^6^A writers, readers, and erasers, and to better understand how chemotherapy itself may feedback into m^6^A pathway regulation, fostering the evolution of resistant subclones.

## Impacts of the m^6^A modification on lncRNA biology and functions

### Impact of the m^6^A modification on lncRNA biogenesis

In breast cancer, systematic mapping of m^6^A-related lncRNAs revealed a positive correlation between lncRNA expression and m^6^A co-expression, underscoring the regulatory significance of this modification for lncRNA biology and tumor behavior. Among m^6^A regulators, RBM15 stands out, exerting widespread effects on the transcriptome by affecting nearly 85% of lncRNAs, thereby acting as a major regulator of lncRNA-mediated cancer pathways (197). Other m^6^A regulators, such as METTL3 and YTHDC2, modulate approximately 7% of lncRNAs each, while the remaining enzymes and reader proteins collectively influence only about 1% of lncRNAs (197). It should be noted, however, that these correlations were derived from RNA expression data, which may not directly reflect actual protein abundance, activity, or functionality within the m^6^A pathway. Therefore, while these findings highlight possible central roles for m^6^A signaling, particularly through RBM15, in shaping the biogenesis and function of lncRNAs as prognostic biomarkers and components influencing immunotherapy response in breast cancer, further studies are needed to confirm these relationships at the protein and functional levels.

### Impact of the m^6^A modification on lncRNA stability

Emerging evidence demonstrates that m^6^A modifications intricately regulate the stability and expression of lncRNAs in breast cancer, thereby shaping tumor growth, drug resistance, and metastatic behavior. Several key studies have revealed that lncRNAs modified by m^6^A can be either stabilized or targeted for decay, in a context-dependent manner, by m^6^A reader proteins. For example, IGF2BP1 is a well-characterized m^6^A reader protein that stabilizes lncRNAs by specifically binding to m^6^A-modified transcripts, protecting them from degradation and enhancing their cellular abundance. IGF2BP1 employs several strategies to stabilize lncRNAs: (i) direct binding to specific elements, such as the coding region instability determinant, which shields transcripts from degradation pathways (104, 198), (ii) recruitment of protective factors (199), (iii) shuttling target RNAs into protective environments such as processing bodies (P-bodies) or stress granules (200), or (iv) incorporation into RNA scaffolds (201).

For example, in the case of MIR210HG, m^6^A methylation creates binding sites for IGF2BP1, which then directly associates with the modified lncRNA and protects it from RNase-mediated degradation. This IGF2BP1-mediated stabilization increases the abundance of MIR210HG, thereby promoting breast cancer progression (188). Furthermore, like IGF2BP1, METTL3-mediated m^6^A methylation stabilizes LINC00958 and drives tumorigenesis *via* the miR-378a-3p/YY1 axis (202). Specifically, m^6^A-dependent stabilization of LINC00958 enhances its interaction with miR-378a-3p, ultimately leading to increased YY1 expression. Conversely, m^6^A reader proteins like YTHDF2 can recognize and bind to m^6^A marks on lncRNAs such as FGF14-AS2, promoting their decay and leading to enhanced RUNX2 translation and osteolytic bone metastasis in breast cancer (203). Moreover, transcriptome-wide mapping has shown that overexpression or dysregulation of m^6^A writers can profoundly reshape the landscape of lncRNA stability and function in breast cancer cells, resulting in an altered TME and disease progression (204). These findings highlight the m^6^A modification as both a stabilizer and a destabilizer of lncRNAs, operating through specific reader and writer proteins to integrate epitranscriptomic signals with oncogenic pathways, highlighting the importance of m^6^A-lncRNA networks in breast cancer pathogenesis and response to treatment.

### Impact of the m^6^A modification on lncRNA action

In breast cancer, the m^6^A modification not only affects the stability and abundance of lncRNAs but also exerts profound control over their functional roles in gene regulation and tumor progression. A growing body of evidence demonstrates that m^6^A methylation can induce local and global changes in lncRNA secondary structure, described above as the “m^6^A switch” (101). In this process, m^6^A marks disrupt base-pairing or promote alternative folding patterns, resulting in the exposure or blocking of specific RNA motifs. This structural remodeling directly influences the ability of lncRNAs to bind regulatory proteins, such as readers, interact with miRNAs, or assemble into molecular scaffolds. Therefore, these m^6^A driven structural changes can dramatically reprogram lncRNA function, connecting epitranscriptomic signals to phenotypic outcomes in breast cancer.

Remarkably, the activity of lncRNAs in cancer-related pathways can depend on a single m^6^A site. For example, methylation at a specific adenosine on HOTAIR is necessary for its proper function as a scaffold and regulator of gene expression, ultimately impacting breast cancer progression (205). Beyond individual sites, global m^6^A methylation orchestrated by METTL3 has been shown to control essential metastatic processes through coordinated lncRNA/miRNA/protein axes. In this respect, METTL3-directed m^6^A on MALAT1 facilitates the repression of miR-26b and subsequent activation of HMGA2, driving metastatic potential in breast cancer cells (206). Additionally, the m^6^A writer KIAA1429 methylates LINC00667, and this m^6^A mark increases LINC00667 RNA stability/accumulation, enabling LINC00667 to sponge miR-556-5p and thereby de-repress KIAA1429, creating a KIAA1429–m^6^A–LINC00667 positive feedback loop that promotes proliferation and migration (207). Collectively, these studies indicate that m^6^A methylation is a key modulator of lncRNA activities in breast cancer, determining their capacity to act as molecular scaffolds, sponges, or regulators within oncogenic or tumor suppressive networks. Understanding these nuanced epitranscriptomic controls offers promising new avenues for therapeutic intervention targeting lncRNA-m^6^A interactions in breast cancer.

### Impact of the m^6^A modification on the ceRNA mechanism of lncRNAs

Recent research highlights how m^6^A modifications promote the ceRNA mechanism in breast cancer, in which lncRNAs and circRNAs function as molecular sponges to sequester miRNAs, thereby shaping post-transcriptional gene regulatory networks. Specifically, m^6^A methylation enhances the expression and stability of ceRNA transcripts, strengthening their ability to interact with and “compete” for miRNA binding. For example, METTL3 installs m^6^A on the lncRNA LINC00958, which increases LINC00958 transcript stability and thereby elevates its expression in breast cancer cells. The accumulated LINC00958 then functions as a ceRNA that sequesters miR-378a-3p, relieving repression of YY1 and promoting tumorigenic phenotypes (202). Furthermore, while m^6^A-dependent upregulation of circMETTL3 promotes breast cancer progression through circRNA–miRNA–mRNA interactions (190). Similarly, WTAP-mediated m^6^A modification of the lncRNA DLGAP1-AS1 increases its RNA stability/abundance, which enhances DLGAP1-AS1’s ability to sponge miR-299-3p. This relieves miR-299-3p–dependent repression of WTAP (*via* the WTAP mRNA 3′UTR), thereby reinforcing a WTAP–DLGAP1-AS1–miR-299-3p–positive feedback loop that promotes chemoresistance and aggressive tumor behavior (39). Moreover, METTL3-mediated m^6^A methylation of the lncRNA WFDC21P increases WFDC21P expression in TNBC cells. Elevated WFDC21P then sponges miR-628-5p to derepress SMAD3, promoting proliferation and metastasis (208).

On the other hand, certain m^6^A-regulated ceRNAs can act as tumor suppressors. In support of this, Fan and Wang, 2021 found that LINC00675 suppresses breast cancer progression by inhibiting miR-513b-5p (209), while upregulation of LINC00520 *via* m^6^A-dependent mechanisms accelerates tumor progression through regulation of the miR-577/POSTN axis and downstream signaling (191). The complexity of the m^6^A modification in ceRNA networks is further exemplified by circBACH2/miR-944/HNRNPC and lncRNA UCA1/miR-375 axes, where m^6^A modifications coordinate the stability, localization, and miRNA sponging activity of ceRNAs (172, 210). Taken together, these observations underscore that m^6^A modification is a central regulator of ceRNA networks, enabling dynamic cooperation between lncRNAs, circRNAs, and miRNAs to drive breast cancer development, metastasis, and treatment resistance.

### Impact of the m^6^A modification on lncRNA function in metastasis

**Figure 4:**
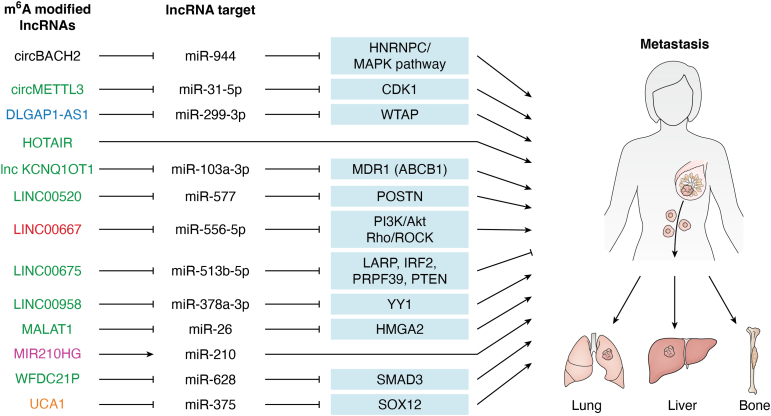
**Schematic representation of m^6^A-modified lncRNAs and their interactions with miRNA targets driving breast cancer metastasis.** This diagram illustrates a panel of m^6^A-modified lncRNAs and circRNAs, such as circBACH2, circMETTL3, DLGAP1-AS1, HOTAIR, KCNQ1OT1, LINC00520, LINC00667, LINC00675, LINC00958, MALAT1, MIR210HG, and WFDC21P, along with their corresponding miRNA targets and downstream genes implicated in promoting or inhibiting metastasis. To facilitate understanding, lncRNAs are color-coded as in Figure. 3 to indicate distinct m^6^A-mediated regulatory mechanisms: *green* represents METTL3/14-induced increased expression through enhanced stability and, in some cases, further stabilization *via* IGF2BP1 or YTHDF protein binding, or increased association between lncRNA and its targets, *red* indicates VIRMA-driven increases in stability, *blue* marks WTAP-mediated stabilization, *pink* highlights IGF2BP1-induced lncRNA stabilization, *orange* is specific to UCA1, which decreases METTL14 expression by recruiting DNA methyltransferase 1 (DNMT1), resulting in reduced stability and expression of the target miRNA, and *black* denotes mechanisms that remain unknown or are not fully characterized for these lncRNAs. Many m^6^A-modified lncRNAs act as molecular sponges, sequestering miRNAs, and derepressing downstream prometastatic gene networks. Gene targets are omitted where validated targets were not reported. Collectively, the epitranscriptomic regulation of these lncRNA-miRNA axes play a central role in promoting breast cancer metastatic progression. lncRNA, long noncoding RNA; m^6^A, N6-methyladenosine; METTL3, methyltransferase like 3; METTL14, methyltransferase like 14; miRNA, microRNA; VIRMA, Vir-like M6A methyltransferase associated; WTAP, Wilms tumor 1–associated protein; YTH, YT521-B homology; YTHDF, YTH m^6^A RNA-binding protein.

The m^6^A modification of lncRNAs serves as a key regulator of metastatic behavior in breast cancer by altering lncRNA stability, abundance, and function. M^6^A-marked lncRNAs interact with specific miRNAs to modulate gene networks that drive invasion, survival, and metastatic progression (Fig. 4). The following sections will further detail how m^6^A-dependent changes in lncRNA biology contribute to core metastatic processes, including migration, invasion, proliferative signaling, and therapeutic resistance, highlighting recently uncovered molecular mechanisms and clinical implications.

### Migration and invasion

The m^6^A modification of lncRNAs is a key epitranscriptomic mechanism driving cellular motility and tissue infiltration. For example, MALAT1 methylation by METTL3 enables it to serve as a molecular scaffold, affecting splicing regulators and chromatin-modifying complexes that tune gene expression profiles toward an invasive phenotype. This m^6^A modification also enhances MALAT1’s function as a ceRNA, allowing it to sponge miR-26b, derepress HMGA2, a key effector of EMT and cytoskeletal dynamics, thereby promoting cell motility and invasiveness (206). Mechanistically, m^6^A deposition induces local changes in MALAT1’s secondary structure (“m^6^A switch” (101)), which exposes miR-26b binding sites and increases MALAT1’s affinity for the miRNA.

LINC00667 is another lncRNA whose role in migration and invasion is amplified by m^6^A methylation. m^6^A methylation of LINC00667 not only increases its abundance but also allows LINC00667 to participate in a positive feedback loop with KIAA1429, sustaining high levels of m^6^A modification in the cell (207). m^6^A-modified LINC00667 can activate promigratory signaling pathways, such as PI3K/AKT and Rho/ROCK, upregulating genes involved in cell junction breakdown and movement through the extracellular matrix (207).

Together, the coordinated m^6^A modification of lncRNAs positions these molecules as master regulators of breast cancer migration, invasion, and metastatic competence. However, it should be noted that m^6^A reader proteins such as IGF2BP can protect pro-EMT lncRNAs from degradation (93), ensuring their continued impact on cellular phenotype. These processes are finely tuned by microenvironmental cues, including hypoxia and inflammation, which modulate the expression and activity of both m^6^A writers and erasers, providing a dynamic adaptation to changing conditions within the tumor and metastatic site. Overall, understanding the distinct roles of m^6^A-writers, readers, and microenvironmental regulation in lncRNA biology provides not only mechanistic insights but also identifies promising targets for therapeutic intervention to impede metastatic progression.

### Growth and proliferation

Uncontrolled proliferation is a hallmark of metastatic breast cancer, and recent evidence demonstrates that m^6^A methylation provides a crucial layer of regulation for the lncRNAs that drive this process. In support of this, the m^6^A epitranscriptomic modification increases the stability and cellular abundance of proproliferative lncRNAs, such as MIR210HG and LINC00958, preventing their degradation by recruiting “reader” proteins like IGF2BP1. IGF2BP1 binds to m^6^A-modified transcripts and shields them from exonuclease activity, effectively extending their half-lives and allowing persistent accumulation (188, 202).

The enhanced stability of these lncRNAs can enable sustained activation of proliferation-promoting genes. For example, MIR210HG has been shown to regulate cell cycle progression and metabolic programs by modulating the expression of metabolic enzymes (211), which can facilitate rapid tumor growth. LINC00958 likewise acts as a ceRNA, sequestering regulatory miRNAs and derepressing downstream targets involved in cell cycle control and biosynthetic pathways, further intensifying the proliferative output of cancer cells (202). The global reach of RBM15, *via* its ability to impact the m^6^A status of a vast majority of lncRNAs in breast cancer, highlights how broad epitranscriptomic stabilization of oncogenic lncRNAs can shift the balance toward unchecked cell division, aiding metastatic colonization and expansion (197).

Furthermore, the level and activity of m^6^A machinery is directly impacted by microenvironmental cues. For example, hypoxic conditions, a common feature of rapidly growing tumors, increase the expression and nuclear activity of m^6^A “writers” (36, 123). This results in greater methylation of lncRNAs associated with stemness and self-renewal, such as NANOG and RIPOR3, reinforcing progenitor-like properties and ensuring that cancer cells retain their capacity for sustained proliferation. These hypoxia-induced changes in m^6^A levels also contribute to metabolic reprogramming and resistance to cellular stress, further increasing tumor aggressiveness and the likelihood of successful metastatic colonization.

Taken together, the m^6^A-dependent stabilization and functional enhancement of oncogenic lncRNAs integrates intrinsic epitranscriptomic mechanisms with extrinsic signals from the TME to drive aggressive breast cancer growth, supporting continuous growth, expansion in adverse niches, and the maintenance of metastatic potential.

### Drug resistance

The m^6^A modification of lncRNAs is increasingly recognized as a key driver of resistance to chemotherapeutic agents and targeted therapies in metastatic breast cancer. Through both direct and network-level effects, m^6^A methylation shapes the fate, function, and interactions of lncRNAs pivotal for chemoresistance.

A key illustration of this regulatory complexity is provided by DLGAP1-AS1. As previously described, m^6^A methylation stabilizes DLGAP1-AS1, enabling it to act as a miR-299-3p sponge. This reduces miRNA-mediated suppression of WTAP, thereby upregulating WTAP and amplifying m^6^A-dependent gene networks that promote cell survival and resistance to adriamycin (39), a first-line treatment for breast cancer.

Another critical axis is defined by KCNQ1OT1, where METTL3-mediated m^6^A addition increases the lncRNA’s stability, enabling it to regulate the miR-103a-3p/MDR1 axis. KCNQ1OT1 sponges miR-103a-3p, which otherwise would inhibit MDR1 (ABCB1) mRNA translation and protein expression. By sequestering miR-103a-3p, KCNQ1OT1 causes MDR1 upregulation, and MDR1 acts as an ATP-dependent drug efflux pump, exporting agents like doxorubicin from the cell and thereby reducing cytotoxic efficacy, which directly promotes multidrug resistance (195).

MALAT1 provides a compelling example of how m^6^A-modified lncRNAs contribute to apoptotic resistance and cell survival in breast cancer. In adriamycin-resistant breast cancer cells, METTL3 increases the m^6^A modification level of MALAT1 and elevates MALAT1 expression. Functionally, m^6^A-modified MALAT1 promotes adriamycin resistance by recruiting E2F1 to the AGR2 promoter and enhancing AGR2 transcription (212). More broadly, lncRNAs can promote apoptotic resistance by sequestering tumor-suppressive miRNAs and by interacting with chromatin or signaling proteins (213, 214, 215), shifting expression toward anti-apoptotic programs (*e.g.*, increased BCL2 and reduced BAX/caspase activity (214, 215)) and by engaging adaptive stress responses such as autophagy and DNA-repair modulation. Taken together, the interplay of m^6^A writers, lncRNAs, miRNAs, and effector proteins constitutes an adaptive gene regulatory network that endows breast cancer cells with increased therapy resistance.

### Conclusions, therapeutic implications, and challenges

Overall, mounting evidence demonstrates that m^6^A methylation serves as a critical regulator of miRNA and lncRNA biogenesis, stability, and function in breast cancer, with far-reaching implications for tumor progression, metastasis, therapy resistance, and clinical outcome. Post-transcriptional m^6^A marks orchestrate fine-tuned networks of gene control through a variety of mechanisms, where it controls miRNA processing and targeting effectiveness, modulating lncRNA expression, stability, and cellular action, and coordinating the crosstalk between noncoding RNAs and protein-coding targets *via* ceRNA networks. Despite these advances, major gaps remain regarding (i) the context-specific effects of individual “writers,” “erasers,” and “readers,” (ii) the functional consequences of m^6^A dynamics in the TME, and (iii) the interplay between m^6^A, immune evasion, and metastatic colonization. At the mechanistic level, how m^6^A influences the stability, decay, and interaction networks of specific miRNAs and lncRNAs, such as MIR210HG, LINC00958, and FGF14-AS2, remains incompletely understood. In some cases, the recruitment of m^6^A reader proteins like IGF2BP1 is known to protect lncRNAs from degradation, but for many transcripts, the biochemical details and structural changes involved are yet to be clarified. Addressing these open questions is a clear direction for future work, which will require high-resolution epitranscriptomic approaches, advanced RNA structure mapping, and functional genomics screens to fully characterize m^6^A-dependent regulatory mechanisms in both physiological and pathological contexts. With ongoing progress, the m^6^A–miRNA/lncRNA axis holds promise both as a source of novel biomarkers for metastasis risk and therapy response and as a foundation for next-generation epitranscriptomic therapies to halt metastatic breast cancer.

The discovery of m^6^A modifications as key modulators of miRNA and lncRNA biogenesis, stability, and function in breast cancer opens multiple avenues for clinical translation and therapeutic innovation. The ability to pharmacologically target m^6^A machinery, such as with emerging METTL3 inhibitors (216), holds promise for disrupting oncogenic epitranscriptomic pathways and reducing metastasis, especially when paired with strategies to restore or inhibit specific miRNAs or lncRNAs that are aberrantly modified in cancer. Importantly, m^6^A-modified noncoding RNAs are increasingly recognized as robust biomarkers. Tissue and serum levels of m^6^A regulators and m^6^A-tagged miRNAs can enhance early cancer detection (217, 218, 219), while m^6^A-lncRNA signatures and hub genes correlate strongly with patients’ prognosis, therapy response, and risk stratification (220). These insights have led to the development and validation of predictive models based on m^6^A-related gene and lncRNA expression for guiding personalized treatment in breast cancer (197). Looking forward, challenges remain in the translation of m^6^A-targeted strategies to routine clinical practice. The complexity of m^6^A-dependent regulatory networks, context-dependent effects, and tissue specificity must be carefully considered in drug design and biomarker development. Nonetheless, ongoing studies and technological advances in high-resolution epitranscriptomic profiling, specific inhibitor development, and RNA modification mapping pave the way for integrating m^6^A biology into precision oncology, ultimately aiming to improve outcomes for patients with metastatic breast cancer.

## Conflict of interest

The authors declare that they have no conflicts of interest with the contents of this article.
